# 2,3-Dihydro­pyrrolo­[2,1-*b*]quinazoline-9(1*H*)-thione

**DOI:** 10.1107/S1600536812021228

**Published:** 2012-05-16

**Authors:** Azizbek O. Nasrullayev, Burkhon Zh. Elmuradov, Kambarali K. Turgunov, Bakhodir Tashkhodjaev, Khusnutdin M. Shakhidoyatov

**Affiliations:** a S. Yunusov Institute of the Chemistry of Plant Substances, Academy of Sciences of Uzbekistan, Mirzo Ulugbek Str. 77, Tashkent 100170, Uzbekistan

## Abstract

In the crystal, mol­ecules of the title compound, C_11_H_10_N_2_S, are connected by C—H⋯N inter­actions around threefold axes. Furthermore, they form stacks along the *c* axis showing π–π inter­actions between pyrimidine rings [centroid–centroid distance = 3.721 (1) Å]. The central ring is essentially planar with an r.m.s. deviation of 0.007 Å. The five-membered ring adopts an envelope conformation with the flap atom deviating by 0.241 (4) Å from the mean plane (r.m.s. deviation = 0.002 Å) through the other four ring atoms.

## Related literature

For the synthesis of 2,3-dihydro-1*H*,9*H*-pyrrolo­[2,1-*b*]quinazolin-9-one and the title compound, see: Abduraza­kov *et al.* (2007 ▶); Shakhidoyatov & Kadyrov (1977 ▶); Elmuradov *et al.* (2010 ▶). For related structures, see Elmuradov *et al.* (2010 ▶); Turgunov *et al.* (1995 ▶).
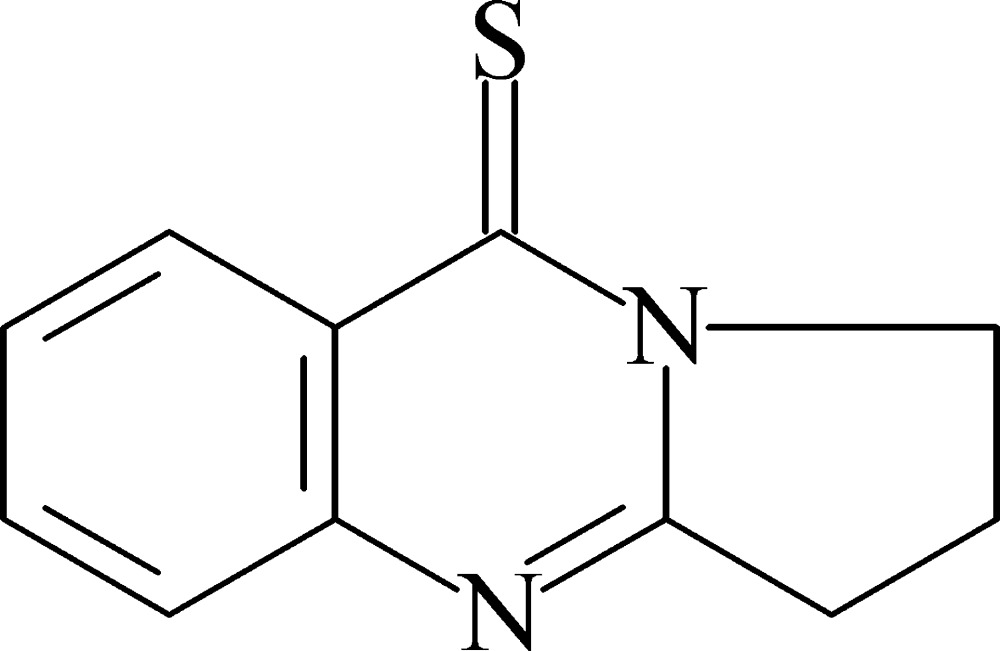


## Experimental

### 

#### Crystal data

C_11_H_10_N_2_S
*M* *_r_* = 202.27
Trigonal, 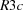

*a* = 26.206 (1) Å
*c* = 7.441 (2) Å
*V* = 4425.5 (12) Å^3^
*Z* = 18
Cu *K*α radiation
μ = 2.57 mm^−1^
*T* = 295 K
0.65 × 0.25 × 0.20 mm

#### Data collection

Oxford Diffraction Xcalibur Ruby diffractometer
Absorption correction: multi-scan (*CrysAlis PRO*; Oxford Diffraction, 2009 ▶) *T*_min_ = 0.601, *T*_max_ = 1.000
5753 measured reflections
1379 independent reflections
1305 reflections with *I* > 2σ(*I*)
*R*_int_ = 0.021

#### Refinement

*R*[*F* ^2^ > 2σ(*F* ^2^)] = 0.029
*wR*(*F* ^2^) = 0.078
*S* = 1.06
1379 reflections
128 parameters
1 restraint
H-atom parameters constrained
Δρ_max_ = 0.16 e Å^−3^
Δρ_min_ = −0.19 e Å^−3^
Absolute structure: Flack (1983 ▶), 501 Friedel pairs
Flack parameter: −0.003 (19)

### 

Data collection: *CrysAlis PRO* (Oxford Diffraction, 2009 ▶); cell refinement: *CrysAlis PRO*; data reduction: *CrysAlis PRO*; program(s) used to solve structure: *SHELXS97* (Sheldrick, 2008 ▶); program(s) used to refine structure: *SHELXL97* (Sheldrick, 2008 ▶); molecular graphics: *XP* (Bruker, 1998 ▶); software used to prepare material for publication: *publCIF* (Westrip, 2010 ▶).

## Supplementary Material

Crystal structure: contains datablock(s) I, global. DOI: 10.1107/S1600536812021228/bt5913sup1.cif

Structure factors: contains datablock(s) I. DOI: 10.1107/S1600536812021228/bt5913Isup2.hkl

Supplementary material file. DOI: 10.1107/S1600536812021228/bt5913Isup3.cml

Additional supplementary materials:  crystallographic information; 3D view; checkCIF report

## Figures and Tables

**Table 1:** Hydrogen-bond geometry (Å, °)

*D*—H⋯*A*	*D*—H	H⋯*A*	*D*⋯*A*	*D*—H⋯*A*
C7—H7*A*⋯N1^i^	0.93	2.61	3.464 (4)	153

Symmetry code: (i) 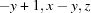.

**Fig. 1.**
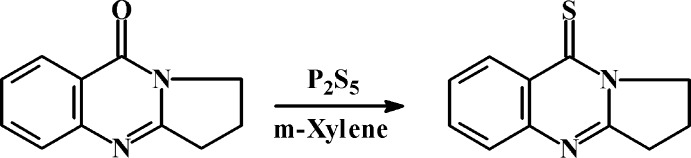
Reaction scheme

**Fig. 2.**
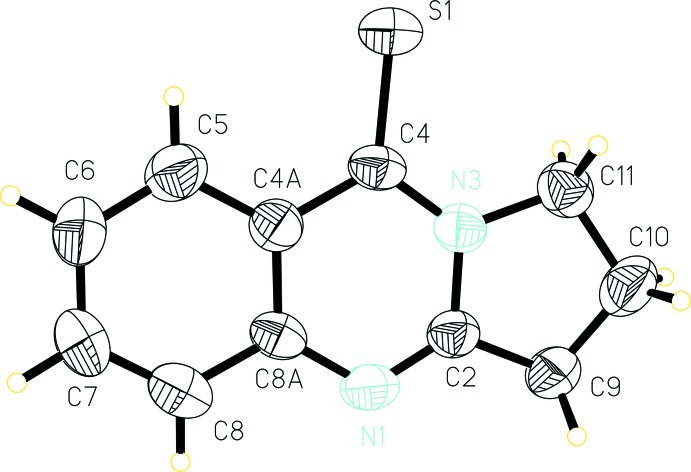
The molecular structure of the title compound with the atom-numbering scheme. Displacement ellipsoids are drawn at the 50% probability level.

## supplementary crystallographic information

### Comment

The title compound was synthesized by the reaction of 2,3-dihydro-1H,9*H*-pyrrolo[2,1-*b*]quinazolin-9-one with phosphorus pentasulfide (Figure 1). X-ray single-crystal diffraction study reveals that the title compound crystallizes in the space group R3c with one molecule in the asymmetric unit. The molecule is almost planar (excluding the atom C10) with r.m.s. deviation of 0.014 Å. The central (pyrimidinic) ring is planar with rms deviations of 0.007Å. Conformation of five-membered (pyrrolic) ring is envelope with deviation of the atom C10 (0.241 (4) Å) from mean plane of other four atoms (rms deviations of 0.002 Å) of the ring. In the structure weak C—H···N interactions (Table 1) are observed. The molecules are stacked along the *c* axis by π–π stacking interactions between pyrimidine rings [centroid-centroid distances = 3.721 (1) Å].

### Experimental

2.5 g (13 mmole) of 2,3-dihydro-1H,9*H*-pyrrolo[2,1]quinazolin-9-one was dissolved in 15 ml *m*-xylene and 2.98 g (13 mmole) of phosphorus pentasulfide were added (Figure 1). Reaction mixture was boiled 2 h and allowed to cool up to room temperature. The precipitate was filtered, flushed with *m*-xylene (3 ml) and 10% NaOH (50 ml) was added, then the precipittate was filtered and washed with water to get neutral medium and was dried. After recrystallization from hexane 1.96 g (72%) the title compound crystals. Suitable for X-ray diffraction crystals was obtained from hexane with m.p. 138 °C

^1^H NMR (400 MHz, CDCl_3_): 8.67 (1*H*, dd, J=8.3, J=1.7, H-8), 7.69 (1*H*, td, J=8.3, J=1.7, H-6), 7.59 (1*H*, dd, J=8.3, J=1.2, H-5), 7.43 (1*H*, td, J=8.3, J=1.2, H-6), 4.47 (2*H*, t, J=7.5, 1-CH_2_), 3.25 (2*H*, t, J=7.9, 3-CH_2_), 2.28 (2*H*, m, 2-CH_2_)

### Refinement

H atoms were positioned geometrically and treated as riding on their C atoms, with C—H distances of 0.93 Å (aromatic)and 0.97 Å (CH_2_) and were refined with *U*_iso_(H)=1.2Ueq(C).

### Crystal data

**Table:**

C_11_H_10_N_2_S	*D*_x_ = 1.366 Mg m^−^^3^
*M**_r_* = 202.27	Melting point: 411 K
Trigonal, *R*3*c*	Cu *K*α radiation, λ = 1.54184 Å
Hall symbol: R 3 -2"c	Cell parameters from 2338 reflections
*a* = 26.206 (1) Å	θ = 3.4–66.8°
*c* = 7.441 (2) Å	µ = 2.57 mm^−^^1^
*V* = 4425.5 (12) Å^3^	*T* = 295 K
*Z* = 18	Prism, yellow
*F*(000) = 1908	0.65 × 0.25 × 0.20 mm

### Data collection

**Table:**

Oxford Diffraction Xcalibur Ruby diffractometer	1379 independent reflections
Radiation source: Enhance (Cu) X-ray Source	1305 reflections with *I* > 2σ(*I*)
Graphite monochromator	*R*_int_ = 0.021
Detector resolution: 10.2576 pixels mm^-1^	θ_max_ = 66.8°, θ_min_ = 3.4°
ω scans	*h* = −28→31
Absorption correction: multi-scan (*CrysAlis PRO*; Oxford Diffraction, 2009)	*k* = −31→31
*T*_min_ = 0.601, *T*_max_ = 1.000	*l* = −8→8
5753 measured reflections	

### Refinement

**Table:**

Refinement on *F*^2^	Hydrogen site location: inferred from neighbouring sites
Least-squares matrix: full	H-atom parameters constrained
*R*[*F*^2^ > 2σ(*F*^2^)] = 0.029	*w* = 1/[σ^2^(*F*_o_^2^) + (0.0613*P*)^2^ + 0.028*P*] where *P* = (*F*_o_^2^ + 2*F*_c_^2^)/3
*wR*(*F*^2^) = 0.078	(Δ/σ)_max_ < 0.001
*S* = 1.06	Δρ_max_ = 0.16 e Å^−^^3^
1379 reflections	Δρ_min_ = −0.19 e Å^−^^3^
128 parameters	Extinction correction: *SHELXL97* (Sheldrick, 2008), Fc^*^=kFc[1+0.001xFc^2^λ^3^/sin(2θ)]^-1/4^
1 restraint	Extinction coefficient: 0.00144 (11)
Primary atom site location: structure-invariant direct methods	Absolute structure: Flack (1983), 501 Friedel pairs
Secondary atom site location: difference Fourier map	Flack parameter: −0.003 (19)

### Special details

**Table:**

Geometry. All e.s.d.'s (except the e.s.d. in the dihedral angle between two l.s. planes) are estimated using the full covariance matrix. The cell e.s.d.'s are taken into account individually in the estimation of e.s.d.'s in distances, angles and torsion angles; correlations between e.s.d.'s in cell parameters are only used when they are defined by crystal symmetry. An approximate (isotropic) treatment of cell e.s.d.'s is used for estimating e.s.d.'s involving l.s. planes.
Refinement. Refinement of *F*^2^ against ALL reflections. The weighted *R*-factor *wR* and goodness of fit *S* are based on *F*^2^, conventional *R*-factors *R* are based on *F*, with *F* set to zero for negative *F*^2^. The threshold expression of *F*^2^ > σ(*F*^2^) is used only for calculating *R*-factors(gt) *etc*. and is not relevant to the choice of reflections for refinement. *R*-factors based on *F*^2^ are statistically about twice as large as those based on *F*, and *R*- factors based on ALL data will be even larger.

### Fractional atomic coordinates and isotropic or equivalent isotropic displacement parameters (Å^2^)

**Table:**

	*x*	*y*	*z*	*U*_iso_*/*U*_eq_	
S1	0.89225 (2)	0.23959 (3)	0.13284 (10)	0.0588 (2)	
N1	0.71400 (8)	0.21624 (8)	0.2807 (3)	0.0562 (5)	
C2	0.72288 (9)	0.17396 (9)	0.2343 (3)	0.0480 (4)	
N3	0.77699 (7)	0.18140 (7)	0.1857 (2)	0.0445 (4)	
C4	0.82758 (8)	0.23443 (9)	0.1852 (3)	0.0451 (4)	
C4A	0.81976 (9)	0.28375 (9)	0.2345 (3)	0.0477 (4)	
C5	0.86723 (10)	0.34181 (10)	0.2392 (4)	0.0614 (5)	
H5A	0.9050	0.3495	0.2105	0.074*	
C6	0.85814 (13)	0.38726 (11)	0.2857 (5)	0.0765 (8)	
H6A	0.8898	0.4256	0.2880	0.092*	
C7	0.80209 (14)	0.37644 (12)	0.3296 (5)	0.0827 (9)	
H7A	0.7964	0.4076	0.3604	0.099*	
C8	0.75527 (11)	0.32025 (12)	0.3276 (5)	0.0746 (7)	
H8A	0.7180	0.3135	0.3581	0.090*	
C8A	0.76270 (10)	0.27240 (9)	0.2803 (3)	0.0528 (5)	
C9	0.67738 (10)	0.11011 (10)	0.2275 (4)	0.0601 (5)	
H9A	0.6436	0.1040	0.1570	0.072*	
H9B	0.6642	0.0947	0.3475	0.072*	
C10	0.70825 (10)	0.08040 (10)	0.1391 (4)	0.0670 (6)	
H10A	0.6999	0.0450	0.2044	0.080*	
H10B	0.6949	0.0697	0.0161	0.080*	
C11	0.77385 (10)	0.12481 (9)	0.1437 (4)	0.0548 (5)	
H11A	0.7934	0.1146	0.2356	0.066*	
H11B	0.7920	0.1267	0.0283	0.066*	

### Atomic displacement parameters (Å^2^)

**Table:**

	*U*^11^	*U*^22^	*U*^33^	*U*^12^	*U*^13^	*U*^23^
S1	0.0415 (3)	0.0673 (4)	0.0720 (3)	0.0306 (2)	0.0040 (2)	−0.0012 (3)
N1	0.0407 (8)	0.0548 (10)	0.0767 (12)	0.0265 (8)	−0.0002 (8)	0.0031 (9)
C2	0.0375 (9)	0.0509 (10)	0.0541 (10)	0.0210 (8)	−0.0041 (8)	0.0011 (9)
N3	0.0422 (8)	0.0457 (9)	0.0480 (8)	0.0238 (7)	−0.0023 (6)	0.0014 (7)
C4	0.0420 (9)	0.0512 (11)	0.0442 (9)	0.0250 (9)	−0.0033 (8)	0.0018 (8)
C4A	0.0444 (10)	0.0472 (10)	0.0541 (10)	0.0249 (8)	−0.0033 (8)	0.0033 (9)
C5	0.0499 (11)	0.0504 (11)	0.0791 (14)	0.0215 (9)	−0.0041 (11)	0.0013 (11)
C6	0.0686 (15)	0.0437 (12)	0.110 (2)	0.0229 (12)	−0.0094 (14)	−0.0012 (13)
C7	0.0838 (17)	0.0545 (13)	0.124 (3)	0.0454 (13)	−0.0099 (18)	−0.0084 (15)
C8	0.0615 (14)	0.0649 (14)	0.112 (2)	0.0429 (12)	−0.0006 (15)	−0.0018 (15)
C8A	0.0485 (11)	0.0482 (11)	0.0667 (12)	0.0279 (9)	−0.0062 (10)	0.0009 (9)
C9	0.0429 (10)	0.0519 (11)	0.0752 (13)	0.0160 (9)	−0.0014 (10)	−0.0002 (11)
C10	0.0616 (14)	0.0464 (11)	0.0850 (16)	0.0211 (10)	−0.0043 (13)	−0.0053 (11)
C11	0.0601 (12)	0.0512 (11)	0.0592 (11)	0.0325 (10)	−0.0004 (11)	−0.0029 (12)

### Geometric parameters (Å, º)

**Table:**

S1—C4	1.6771 (18)	C6—H6A	0.9300
N1—C2	1.288 (3)	C7—C8	1.366 (4)
N1—C8A	1.384 (3)	C7—H7A	0.9300
C2—N3	1.380 (3)	C8—C8A	1.406 (3)
C2—C9	1.493 (3)	C8—H8A	0.9300
N3—C4	1.359 (3)	C9—C10	1.524 (3)
N3—C11	1.477 (3)	C9—H9A	0.9700
C4—C4A	1.453 (3)	C9—H9B	0.9700
C4A—C5	1.404 (3)	C10—C11	1.520 (3)
C4A—C8A	1.413 (3)	C10—H10A	0.9700
C5—C6	1.371 (4)	C10—H10B	0.9700
C5—H5A	0.9300	C11—H11A	0.9700
C6—C7	1.388 (4)	C11—H11B	0.9700
			
C2—N1—C8A	116.55 (17)	C7—C8—H8A	119.6
N1—C2—N3	124.32 (18)	C8A—C8—H8A	119.6
N1—C2—C9	125.92 (19)	N1—C8A—C8	118.8 (2)
N3—C2—C9	109.75 (18)	N1—C8A—C4A	122.71 (18)
C4—N3—C2	123.61 (16)	C8—C8A—C4A	118.5 (2)
C4—N3—C11	124.21 (16)	C2—C9—C10	104.87 (19)
C2—N3—C11	112.11 (16)	C2—C9—H9A	110.8
N3—C4—C4A	114.17 (16)	C10—C9—H9A	110.8
N3—C4—S1	120.86 (15)	C2—C9—H9B	110.8
C4A—C4—S1	124.97 (16)	C10—C9—H9B	110.8
C5—C4A—C8A	119.56 (19)	H9A—C9—H9B	108.8
C5—C4A—C4	121.83 (19)	C11—C10—C9	106.58 (18)
C8A—C4A—C4	118.61 (18)	C11—C10—H10A	110.4
C6—C5—C4A	120.2 (2)	C9—C10—H10A	110.4
C6—C5—H5A	119.9	C11—C10—H10B	110.4
C4A—C5—H5A	119.9	C9—C10—H10B	110.4
C5—C6—C7	120.5 (2)	H10A—C10—H10B	108.6
C5—C6—H6A	119.7	N3—C11—C10	104.32 (18)
C7—C6—H6A	119.7	N3—C11—H11A	110.9
C8—C7—C6	120.4 (2)	C10—C11—H11A	110.9
C8—C7—H7A	119.8	N3—C11—H11B	110.9
C6—C7—H7A	119.8	C10—C11—H11B	110.9
C7—C8—C8A	120.9 (2)	H11A—C11—H11B	108.9

### Hydrogen-bond geometry (Å, º)

**Table:**

*D*—H···*A*	*D*—H	H···*A*	*D*···*A*	*D*—H···*A*
C7—H7*A*···N1^i^	0.93	2.61	3.464 (4)	153

Symmetry code: (i) −*y*+1, *x*−*y*, *z*.
